# Optimizing agricultural sustainability: enriched organic formulations for growth, yield, and soil quality in a multi-crop system

**DOI:** 10.3389/fpls.2024.1398083

**Published:** 2024-06-19

**Authors:** Kamal Garg, Shiva Dhar, Vinod Kumar Sharma, Elisa Azura Azman, Rajendra Prasad Meena, Mohammad Hashim, Dileep Kumar, Ghous Ali, Vetrivel Karunakaran, Yogesh Kumar, Sonal Athnere, Sourabh Kumar, Hari Om, Mangal Deep Tuti, Babu Lal Meena, Birendra Kumar, Vijendra Kumar Meena, Sanjeev Kumar

**Affiliations:** ^1^ ICAR-Indian Agricultural Research Institute, New Delhi, India; ^2^ ICAR- National Dairy Research Institute, Karnal, Haryana, India; ^3^ Department of Crop Science, Faculty of Agriculture, Universiti Putra Malaysia, Serdang, Malaysia; ^4^ ICAR-Vivekananda Parvatiya Krishi Anusandhan Sansthan, Almora, Uttarakhand, India; ^5^ ICAR-Indian Institute of Pulses Research, Kanpur, Uttar Pradesh, India; ^6^ ICAR-Indian Institute of Sugarcane Research, Lucknow, Uttar Pradesh, India; ^7^ ICAR-Central Sheep and Wool Research Institute, Arid Regional Campus (ARC), Bikaner, Rajasthan, India; ^8^ ICAR-Krishi Vigyan Kendra Needamangalam, Thiruvarur, Tamil Nadu, India; ^9^ Chaudhary Charan Singh Haryana Agricultural University, College of Agriculture, Rewari, Haryana, India; ^10^ Department of Agronomy, Maharana Pratap University of Agriculture and Technology, Uadipur, Rajasthan, India; ^11^ Department of Agronomy, Veer Kunwar Singh College of Agriculture, Buxar, Bihar, India; ^12^ Department of Agronomy, Bihar Agricultural University, Bhagalpur, Bihar, India; ^13^ ICAR-Indian Institute of Rice Research, Hyderabad, Telangana, India; ^14^ ICAR- Central Soil Salinity Research Institute, Karnal, India

**Keywords:** farmyard manure, microbial population, paddy husk ash, potato peel compost, soil enzymatic activity, yield

## Abstract

Utilizing agricultural and industrial wastes, potent reservoirs of nutrients, for nourishing the soil and crops through composting embodies a sustainable approach to waste management and organic agriculture. To investigate this, a 2-year field experiment was conducted at ICAR-IARI, New Delhi, focusing on a pigeon pea–vegetable mustard–okra cropping system. Seven nutrient sources were tested, including a control (T_1_), 100% recommended dose of nitrogen (RDN) through farmyard manure (T_2_), 100% RDN through improved rice residue compost (T_3_), 100% RDN through a paddy husk ash (PHA)–based formulation (T_4_), 75% RDN through PHA-based formulation (T_5_), 100% RDN through a potato peel compost (PPC)–based formulation (T_6_), and 75% RDN through PPC-based formulation (T_7_). Employing a randomized block design with three replications, the results revealed that treatment T_4_ exhibited the significantly highest seed (1.89 ± 0.09 and 1.97 ± 0.12 t ha^−1^) and stover (7.83 ± 0.41 and 8.03 ± 0.58 t ha^−1^) yield of pigeon pea, leaf yield (81.57 ± 4.69 and 82.97 ± 4.17 t ha^−1^) of vegetable mustard, and fruit (13.54 ± 0.82 and 13.78 ± 0.81 t ha^−1^) and stover (21.64 ± 1.31 and 22.03 ± 1.30 t ha^−1^) yield of okra during both study years compared to the control (T_1_). Treatment T_4_ was on par with T_2_ and T_6_ for seed and stover yield in pigeon pea, as well as okra, and leaf yield in vegetable mustard over both years. Moreover, T_4_ demonstrated notable increase of 124.1% and 158.2% in NH_4_-N and NO_3_-N levels in the soil, respectively, over the control. The enhanced status of available nitrogen (N) and phosphorus (P) in the soil, coupled with increased soil organic carbon (0.41%), total bacteria population (21.1%), fungi (37.2%), actinomycetes (44.6%), and microbial biomass carbon (28.5%), further emphasized the positive impact of T_4_ compared to the control. Treatments T_2_ and T_6_ exhibited comparable outcomes to T_4_ concerning changes in available N, P, soil organic carbon, total bacteria population, fungi, actinomycetes, and microbial biomass carbon. In conclusion, treatments T_4_ and T_6_ emerge as viable sources of organic fertilizer, particularly in regions confronting farmyard manure shortages. These formulations offer substantial advantages, including enhanced yield, soil quality improvement, and efficient fertilizer utilization, thus contributing significantly to sustainable agricultural practices.

## Introduction

The growing global population accentuates the urgency of ensuring food sustainability, safety, and security while lessening the strain on economic, environmental, and Earth resources (Shubbar et al., 2024). Conventional farming, relying on input-intensive practices, poses long-term challenges affecting production systems, biodiversity, food quality, environmental sustainability, greenhouse gas emissions, and human health (Mbow et al., 2020). In response, efforts over the past two decades have focused on exploring sustainable alternatives aligned with the United Nations Sustainable Development Goals, promoting eco-friendly living and environmental stewardship (Imran et al., 2022).

Organic farming, employing natural methods for crop cultivation and ecologically sound fertilization and pest control, emerges as a pivotal approach (Chatterjee et al., 2017). Various organic sources, including farmyard manure (FYM), paddy husk ash (PHA), and potato peel compost (PPC), derived from agricultural and industrial waste, significantly contribute to sustainable practices. FYM, composed of decomposed dung, urine, litter, and leftover fodder, enables gradual nutrient release, aligning with plant requirements (De et al., 2021). Similarly, PHA, a byproduct of rice milling, and potato peel waste from the food processing industry present disposal challenges, motivating their exploration for agricultural applications (Sharma et al., 2015).

While previous research has examined organic farming practices, our study uniquely targets the utilization of these significant industrial byproducts, which are often overlooked in traditional agricultural practices. By repurposing PHA and potato peel waste, we aim to address critical disposal challenges while simultaneously exploring their potential as enriched organic formulations in agriculture.

Composting, a microbial-mediated process accelerating waste breakdown, transforms complex materials into forms suitable for agricultural use (Zhang et al., 2016; Bhattacharjya et al., 2021). This study focuses on PHA and potato peel waste, significant global industrial wastes. Recycling these materials in agriculture not only addresses disposal challenges but also holds potential for reducing reliance on commercial fertilizers (Ayilara et al., 2020). PHA, abundantly produced in rice-growing countries, poses a disposal challenge due to its sheer volume, exacerbating air pollution, and greenhouse gas emissions and causing substantial nutrient loss (Bhuvaneshwari et al., 2019).

Similarly, potato peel waste, a byproduct of the food processing industry, contains substantial starch and nutrients (Joshi et al., 2020). Utilizing this waste in composting not only enriches soil microbes but also tackles the disposal challenges faced by the potato industry (Ezejiofor et al., 2014). By exploring these organic amendments, our research aims to contribute to ongoing efforts in optimizing agricultural sustainability, aligning with the global push toward environmentally friendly practices.

Our hypothesis posits that enriched organic formulations from agricultural waste will foster plant growth and improve soil health compared to conventional production systems. To substantiate this, our research objectives encompass a comprehensive assessment of soil quality through measuring, enzymatic activity, microbial biomass, community composition, and various physical and chemical properties. Additionally, we seek to evaluate the impact of these organic formulations on nutrient accumulation in the soil profile and their influence on the growth and yield of crops, namely, pigeon pea, veg mustard, and okra, grown in rotation.

This study comprehensively assesses the short-term effects of organic formulations on various parameters, including soil quality. The investigation extends its focus to examine how these variables influence the growth and yield of rotationally cultivated pigeon pea–veg mustard–okra crops. Aligned with the overarching goal of advancing agricultural sustainability, this research seeks to unravel the advantages associated with employing enhanced organic formulations derived from agricultural waste in a multi-crop system. By delving into soil quality, nutrient dynamics, and crop performance systematically, the research aims to contribute valuable insights and propose practical solutions to global challenges in both food production and waste management.

## Materials and methods

### Experimental site description

The field experiment was conducted over two consecutive years (2020 and 2021) at the research farm of the Indian Agricultural Research Institute (IARI) in New Delhi (28.38° N, 77.09° E), situated at an elevation of 228.6 m above mean sea level. The site experiences a semi-arid to sub-tropical climate, characterized by extreme cold and hot conditions. Total rainfall during the July–May periods was 913.6 mm in 2020–2021 and 1682.9 mm in 2021–2022. In June, the hottest month, mean maximum temperatures range between 40°C and 45°C, while mean minimum temperatures in January, the coldest month, can drop as low as 1.20°C.

The soil at the experimental site exhibits a sandy clay loam texture (sand 63.8%, silt 12.2%, and clay 24.0%) with a pH of 8.1 and electrical conductivity (EC) of 0.36 dS m^-1^ in the top 15 cm. Initial soil analysis revealed low organic carbon content (0.37%) and available nitrogen (206.2 kg ha^-1^), and medium levels of available phosphorus (13.6 kg ha^-1^) and potassium (236.0 kg ha^-1^). A comprehensive overview of the physio-chemical and biological properties of the soil, as determined in the initial soil examination, is presented in **Table 1**.

**Table 1:** Initial status of soil physico-chemical and biological properties at the experimental site.

Sr. no.	Particulars	Values		Method used and reference
		2020–2021	2021–2022	
**I**	**Physical properties**			
	**Mechanical composition**			
	1. Sand (%)	63.8	63.5	Hydrometer method (Bouyoucos, 1962)
	2. Silt (%)	17.2	16.8	
	3. Clay (%)	19.0	19.7	
	Textural class	Sandy clay loam		Triangular method
	B.D. (0–15 cm layer) (Mg m^-3^)	1.54	1.51	Core sampler (Bodman, 1942)
	1. Water stable aggregates (%)	47.1	49.2	Wet sieving technique (Haynes, 1993)
**II**	**Chemical properties**			
	pH (1:2.5) soil: water ratio	8.1	7.9	Elico pH meter (Piper, 1950)
	EC (dS m^−1^) (1:2) soil: water ratio	0.36	0.35	Solubridge method (Richards, 1954)
	Organic carbon (%)	0.37	0.39	Walkley and Black (Jackson, 1973)
	Available N (kg ha^−1^)	206.2	211.8	Alkaline permanganate method Subbiah and Asija (1956)
	NO_3_-N (mg kg^−1^)	10.4	11.0	(Bremner and Keeney, 1966)
	NH_4_-N (mg kg^−1^)	6.8	7.6	
	Available P (kg ha^−1^)	13.6	14.2	Olsen’s method (Olsen, 1954)
	Available K (kg ha^−1^)	236.0	239.2	Flame photometric method (Jackson, 1973)
**III**	**Biological properties**			
	MBC (µg MBC g^−1^ soil)	177.2	192.6	Nunan et al. (1998)
	DHA activity (µg TPF g^−1^ soil day^−1^)	144.2	149.4	Casida et al. (1964)
	AP activity (μg PNP g^−1^ soil hr^−1^)	55.6	60.8	Tabatabai and Bremner (1969)
**IV**	**Microbial community composition**			
	Bacteria (×10^5^ cfu)	30.9	32.2	Allen (1958)
	Fungi (×10^2^ cfu)	15.5	17.2	Martin (1950)
	Actinomycetes (×10^2^ cfu)	13.5	15.0	Allen (1958)

### Experimentation design and crop management

The experiment employed a completely randomized block design with three replications, encompassing seven nutrient sources tested across each crop season. The treatments included: T_1_ (control), T_2_ [100% recommended dose of nitrogen (RDN) through FYM], T_3_ [100% RDN through improved rice residue compost (RRC)], T_4_ (100% RDN through PHA-based formulation, T_5_ (75% RDN through PHA-based formulation), T_6_ (100% RDN through PPC-based formulation, and T_7_ (75% RDN through PPC-based formulation). Each treatment had a plot size of 5.0 m × 4.5 m. The experiment involved the cultivation of Pigeon pea variety “Pusa Arhar 16,” vegetable mustard “Pusa sag 1,” and okra variety “Pusa Bhindi 5.” The RDN for pigeon pea, vegetable mustard, and okra were 30, 80, and 100 kg N ha^−1^, respectively.

Prior to crop sowing, organic formulations were applied based on RDN and the nutrient requirements of each crop in their respective treatments. The nutrient composition and detailed descriptions of the organic formulations, uniformly applied across all crops in both experimental years, are comprehensively presented in **Tables 2**, **3**. Farmyard manure, rice residue compost, and PPC contained 0.72%, 0.55%, and 0.61% N during 2020–2021, and 0.70%, 0.58%, and 0.59% N (on an oven-dry weight basis) during 2021–2022, respectively.

**Table 2:** Chemical and nutrient composition of organic manures/formulations used in the experiment.

Parameters	Nutrient sources											
	FYM		RRC		PHA		PPC		PHA-based formulation		PP-based formulation	
	2020	2021	2020	2021	2020	2021	2020	2021	2020	2021	2020	2021
pH	8.1	8.0	7.7	7.6	7.8	7.7	7.8	7.9	7.9	7.8	8.0	8.0
EC (ds m^−1^)	3.75	3.76	3.82	3.84	3.87	3.85	3.84	3.86	3.76	3.77	3.77	3.78
TOC (%)	15.42	15.84	13.24	13.96	4.66	4.72	11.56	11.42	14.34	14.73	14.65	14.96
Total N (%)	0.72	0.70	0.55	0.58	0	0	0.61	0.59	0.65	0.63	0.70	0.68
Total P (%)	0.25	0.27	0.22	0.21	0.20	0.17	0.28	0.32	0.25	0.26	0.26	0.28
Total K (%)	0.52	0.49	1.27	1.34	1.66	1.75	0.85	0.90	0.63	0.62	0.59	0.57
Fe (mg kg^−1^)	708.6	778.8	501.2	562.6	1252.4	1298.4	902.8	908.2	1663.0	1730.8	1838.6	1872.7
Mn (mg kg^−1^)	341.7	328.6	232.5	254.3	298.4	312.5	284.0	265.4	327.4	317.0	330.2	316.0
Cu (mg kg^−1^)	36.2	40.5	25.6	30.5	38.4	43.8	55.8	47.5	34.4	38.8	40.7	41.9
Zn (mg kg^−1^)	127.5	115.4	81.7	93.4	98.5	105.5	90.4	102.8	120.8	111.1	122.1	114.9
Bacteria (×10^5^ cfu)	60	61.4	51.3	50.2	14.0	15.2	46.0	48.2	55.8	57.1	58.5	59.8
Fungi (×10^2^ cfu)	43.1	42	36.0	38.2	12.0	11.3	33.0	35.7	40.2	39.9	41.6	41.0
Actinomycetes (×10^2^ cfu)	35	37.5	29.2	30.0	9.0	9.4	26.4	25.0	32.5	32.0	33.4	35.0

**Table 3:** Quantity of organic formulations applied in different crops during both the years of study.

Crop	Year	Input applied (kg ha^−1^)						
		Control	FYM	RRC	PHA-F (100% RDN)	PHA-F (75% RDN)	PP-F (100% RDN)	PP-F (75% RDN)
Pigeon pea	2020	–	4167	5455	4630	3472	4317	3238
	2021	–	4286	5172	4762	3571	4446	3334
Veg. mustard	2020–2021	–	11111	14545	12346	9259	11512	8634
	2021–2022	–	11429	13793	12698	9524	11855	8891
Okra	2020–2021	–	13889	18182	15432	11574	14390	10792
	2021–2022	–	14286	17241	15873	11905	14818	11114

Thinning was conducted at 10 days after sowing (DAS) to achieve the desired plant population, while gap filling was performed at 20 DAS to maintain a plant-to-plant distance of 20 cm in rows. At 20 DAS, one-hand weeding and hoeing were carried out in each plot to minimize weed growth and intensity. Five randomly chosen plants in each plot were tagged to record growth and yield attributing parameters. Harvesting of pigeon pea occurred when pods turned brown and grains attained relative hardness, with a moisture content of 75%–80%. After drying, bundles were threshed using a Pullman thresher, and grains were cleaned, weighed, and converted to a hectare basis.

In vegetable mustard, green leaf yield was recorded after each cutting of leaves from the five selected and tagged plants, and the average leaf yield per hectare was calculated. Similarly, in okra, fruits were manually picked when green, tender, and of marketable size. After each picking, harvested fruits were weighed, and the combined total was calculated at the end to determine the yield in t ha^−1^. Immediately following the last picking of green pods, plants from the net plot were harvested, and the fresh weight was recorded, with the green stover yield expressed in t ha^−1^.

### Preparation of organic formulations

FYM was prepared by aerobic windrow method in biomass utilization unit, IARI New Delhi. On an average, well-decomposed FYM contains approximately 5–6 kg N, 1.2– 2.0 kg P and 5–6 kg K per tonne, respectively. Rice residue compost was prepared by windrow composting method. In this method, rice residue was thoroughly chopped and placed in windrows to hasten rate of decomposition then windrows were turned periodically. During turning, chopped residue was moistened with spray of water and cow dung slurry on the windrow, which speed up the composting process. It takes about 6–8 weeks for complete uniform compost. Rice husk is produced during milling of rice. About 0.20 t of rice husk is produced from 1 ton of paddy and from 1 ton of rice husk, it generates approximately 0.25 ton of ash after burning, depending upon variety and climatic conditions. Recycling PHA in agriculture can address disposal challenges while also reducing the need for commercial fertilizer application. So, in this experiment, PHA-based formulation was made by mixing PHA with FYM in 20:80 ratio on weight basis. Potato peel waste is byproduct of food processing industry. Potato peel contains large amount of starch and nutrients, which can be supplemented with microbes in soil to enrich manure with nitrogen content. It can be used as an alternate source of manure for crops and to reduce environmental pollution. Therefore, potato peel-based formulation was made by mixing potato peel with FYM in 20:80 ratio on volume basis.

### Soil sampling and analysis

The multiple soil samples were collected from different locations across each plot of the experimental field in a zigzag pattern using a core auger from the 0–15 cm soil profile initially. Additionally, at the end of the experimental period, 15 samples were collected from each plot. After removing all stubble, residues, and root biomass, a composite soil sample of approximately 500 g was obtained from each plot. Subsequently, these samples were then air dried, ground, and passed through 2 mm mesh sieve and preserved in air-tight polythene containers for subsequent chemical analysis. The rhizosphere soil samples were collected from the plant roots using a core auger sampler to measure microbial counts and enzymatic activities.

Bulk density (BD) of soil was assessed using core sampler method given by Bodman (1942). Water stable aggregates (WSAs) were quantified using wet sieving technique (Haynes, 1993). pH of the soil water suspension (1:2) was measured potentiometrically using Elico pH meter (Piper, 1950). EC was measured by conductivity bridge method (Jackson, 1973). Microbial biomass carbon (MBC) was estimated using chloroform fumigation method as given by Nunan et al. (1998). Dehydrogenase activity (DHA) was determined according to method given by Casida et al. (1964). Alkaline phosphatase (AP) was assessed using method given by Tabatabai and Bremner (1969). Total bacteria, fungi, and actinomycetes population counts were assessed by using the Allen (1958), Martin (1950) and Allen (1958) methods, respectively. Organic carbon (OC) content in soil samples were assessed using Walkley and Black method (Jackson, 1973). The available N in soil was determined using alkaline potassium permanganate (KMnO_4_) method given by Subbiah and Asija (1956). The available P content in soil was quantified using Olsen’s method as proposed by Olsen (1954). Available K was estimated using neutral ammonium acetate extraction method as described by Jackson (1973). NH_4_^+^-N was analyzed via steam distillation with MgO in a micro-Kjeldahl system, while NO_3_^-^N after reduction with Devarda’s alloy followed by distillation (Bremner and Keeney, 1966). The N content in various plant parts was determined by using the modified Kjeldahl method (Subbiah and Asija, 1956). The apparent N budgeting was done based on initial N content in soils, total N content added through different formulations during two cropping cycle, biological nitrogen fixation value of 170 kg ha^−1^ was utilized for pigeon pea as calculated by Adu-Gyamfi et al. (1997) for one cropping cycle and total N uptake by crop plants and available N content in soil after completion of the two cropping cycles.

### Statistical analysis

The data collected over a two-year period were subjected to statistical analysis utilizing the *F*-test, following the methodology outlined by Gomez and Gomez (1984). To ascertain the significance of differences between treatment means, the standard deviation (SD) and LSD values, or Duncan’s Multiple Range Test at a significance level of *p* ≤ 0.05, were employed, and the results are graphically represented using error bars. The correlation analysis was performed using the JASP software version 0.18.3.0.

## Results

### Growth attributes

#### Pigeon pea

**Figure 1:**
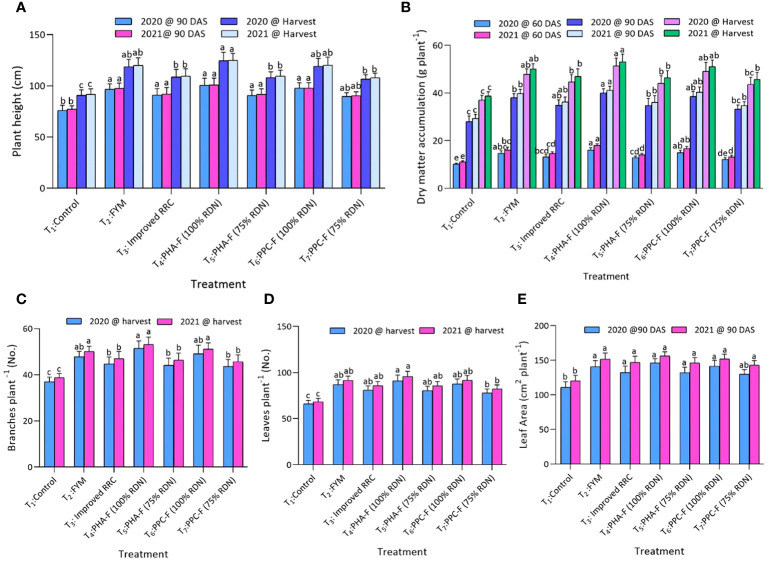
Impact of enriched organic formulations on growth attributes of pigeon pea: **(A)** plant height, **(B)** dry matter accumulation, **(C)** Branches per plant, **(D)** leaves per plant, and **(E)** leaf area during 2020 and 2021. The data represents the mean values across treatments (T_1_–T_7_) with error bars indicating standard deviation. Statistical significance was assessed using one-way ANOVA, with differences considered significant at *p* ≤ 0.05. Similar letter indicates the treatments are at par with each other at *p* ≤ 0.05.

Significant variations were observed in growth attributes of pigeon pea due to application of enriched organic formulations. Treatments T_2_, T_3_, T_4_, T_5,_ and T_6_ also recorded significant superiority over control (T_1_) for all the growth attributes. A significant maximum increase in plant height at 90 DAS (100.7 cm and 100.9 cm) and at harvest stage (124.6 cm and 124.9 cm) recorded, when subject to treatment T_4_ in contrast to control treatment (T_1_) during years 2020 and 2021. In addition, treatment T_4_ exhibited significantly maximum dry matter accumulation (DMA) per plant at 60 DAS (16.1 g and 18.0 g), 90 DAS (40.0 g and 41.1 g) and at harvest (51.4 g and 53.1 g) and number of branches per plant (17.8 and 17.9) at harvest and found significantly superior over treatment T_7_ and T_1_ during 2020 and 2021. Likewise, significant (*p* ≤ 0.05) increased leaf area at 90 DAS (146.1 cm^2^ and 156.2 cm^2^) and leaves per plant (91.0 and 95.1) were observed under T_4_ and found noticeably higher over T_7_ and T_1_ treatments during 2020 and 2021. Treatments T_6_ and T_2_ recorded comparable values with treatment T_4_ for different growth attributing characters (**Figures 1A–E**).

#### Vegetable mustard

**Figure 2:**
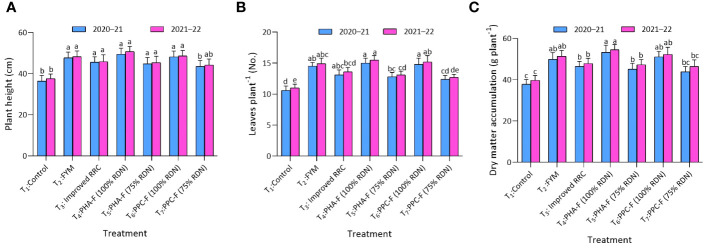
Impact of enriched organic formulations on growth attributes of vegetable mustard: **(A)** plant height, **(B)** leaves per plant, and **(C)** dry matter accumulation during 2020–2021 and 2021–2022 at harvest. The data represent the mean values across treatments (T_1_–T_7_) with error bars indicating standard deviation. Statistical significance was assessed using one-way ANOVA, with differences considered significant at *p* ≤ 0.05. Similar letter indicates the treatments are at par with each other at *p* ≤ 0.05.

Significant (*p* ≤ 0.05) superiority was recorded under treatment T_4_ in the plant height (49.5 and 50.7 cm) in vegetable mustard to the extent of 36.0% and 34.8% over control (T_1_) during year 2020–2021 and 2021–2022. Treatment T_2_, T_3_, T_5,_ and T_6_ found at par with treatment T_4_ and significantly superior over T_1_ for plant height. Similarly, T_4_ asserting considerably (*p* ≤ 0.05) enhanced number of leaves per plant (15.0 and 15.5) and DMA (53.3 g and 54.5 g) at harvest over rest of the treatments and exhibited statistical parity with T_6_ and T_2_ during 2020–2021 and 2021–2022. Further treatment T_3_ and T_5_ also recorded significant superiority over T_1_ for leaves per plant and DMA during year 2020–2021 and 2021–2022 (**Figures 2A–C**).

#### Okra

**Figure 3:**
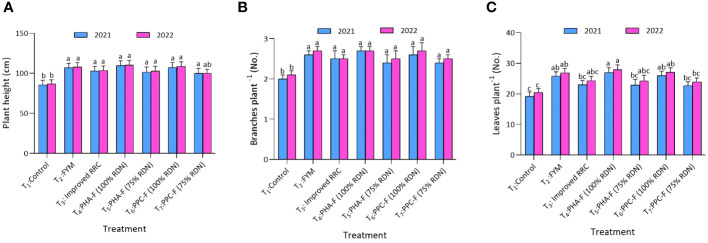
Impact of enriched organic formulations on growth attributes of okra: **(A)** plant height, and **(B)** branches per plant, during 2021 and 2022 at harvest and **(C)** leaves per plant at 60 days after sowing. The data represent the mean values across treatments (T_1_–T_7_) with error bars indicating standard deviation. Statistical significance was assessed using one-way ANOVA, with differences considered significant at *p* ≤ 0.05. Similar letter indicates the treatments are at par with each other at *p* ≤ 0.05.

Application of treatment T_4_ significantly (*p* ≤ 0.05) enhanced the plant height (109.5 and 110.0 cm) and number of branches (2.7 and 2.8) at harvest over the control (T_1_) and exhibited statistical parity with remaining treatments during 2021 and 2022 (**Figures 3A–C**). Likewise, treatment T_4_ recorded the maximum leaves at 60 DAS (27.0 and 27.9) over control to the tune of 40.6 and 36.8%, respectively, and exhibited statistically similarity with T_6_ and T_2_ during 2021 and 2022. Furthermore, treatment T_4_ recorded maximum number of leaves per plant (27.0 and 27.9) and found at par with treatment T_2_ and T_6_ during 2020 and 2022 at 60 DAS. Treatment T_2_ and T_6_ also found at par with T_3_ and T_5_ for leaves per plant at 60 DAS during both the years. Treatment T_1_ recorded lowest leaves per plant (19.2 and 20.4) and found at par with treatment T_7_ during both the years of experiment.

### Yield attributes and yield

#### Pigeon pea

**Figure 4:**
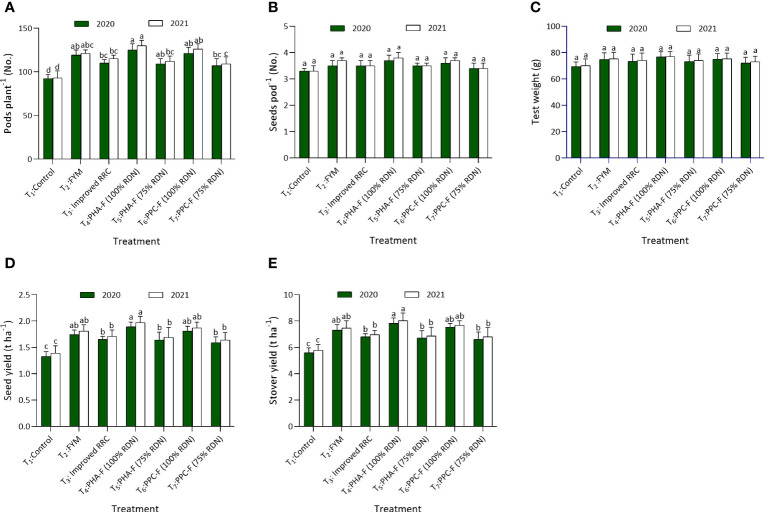
Impact of enriched organic formulations on yield attributes and yield of pigeon pea: **(A)** pods per plant, **(B)** seeds per pod, and **(C)** test weight, **(D)** Seed yield and **(E)** stover yield during 2020 and 2021 at harvest. The data represents the mean values across treatments (T_1_–T_7_) with error bars indicating standard deviation. Statistical significance was assessed using one-way ANOVA, with differences considered significant at *p* ≤ 0.05. Similar letter indicates the treatments are at par with each other at *p* ≤ 0.05.

Significant variations were observed in pods per plant over control when applied with enriched organic formulations. The highest number of pods per plant was found with the application of T_4_ (124.7 and 130.2), which recorded statistical parity with T_6_ and T_2_ during 2020 and 2021. The seeds per pod and 1000 grain weight did not vary noticeably with the use of different enriched organic formulations. Further, seed (1.89 and 1.97 t ha^−1^) and stover (7.83 and 8.03 t ha^−1^) yield was superior under T_4_ being comparable to T_6_ and T_2_ exhibited significant increase of 41.8% and 42.0% higher seed yield over T_1_ treatment (control) during 2020 and 2021, respectively. Treatment T_5_ found at par with T_7_ and recorded 23.0% and 21.6% higher seed yield and 20.3% and 18.9% stover yield compared to control treatment, respectively, during 2020 and 2021. Harvest index of pigeon pea recorded non substantial difference due to different enriched organic formulations as depicted in **Figures 4A–E**.

#### Vegetable mustard

**Figure 5:**
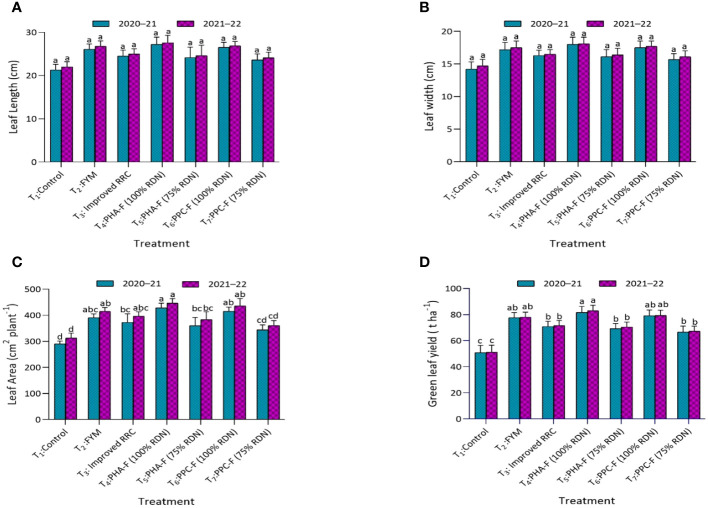
Impact of enriched organic formulations on yield attributes and yield of vegetable mustard: **(A)** leaf length, **(B)** leaf width, **(C)** leaf area, and **(D)** Green leaf yield during 2020–2021 and 2021–2022 at harvest. The data represents the mean values across treatments (T_1_–T_7_) with error bars indicating standard deviation. Statistical significance was assessed using one way ANOVA, with differences considered significant at *p* ≤ 0.05. Similar letter indicates the treatments are at par with each other at *p* ≤ 0.05.

In vegetable mustard, no significant variations were observed in leaf length and leaf with due to application of different treatments. The maximum leaf length and width were recorded under treatment T_4_ followed by T_2_ and T_6_ treatments. Treatment T_4_ was found comparable with T_6_ and T_2_ and demonstrated a significant (*p* ≤ 0.05) enhancement in green leaf yield (81.57 and 82.97 t ha^−1^) of vegetable mustard to the tune of 60.3% and 61.7% during 2020–2021 and 2021–2022, respectively, over control treatment (T_1_). Similarly, application of T_5_ results in 36.1% and 37.6% higher green leaf yield over control treatment (T_1_), respectively, during both the years (**Figures 5A–D**).

#### Okra

**Figure 6:**
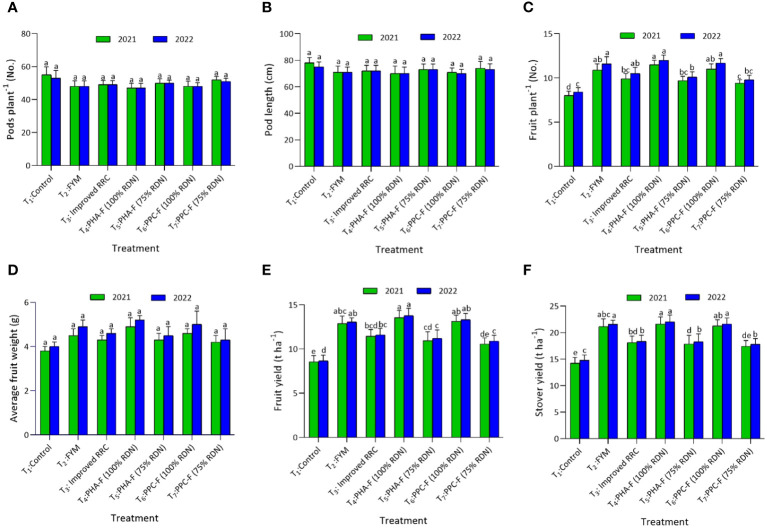
Impact of enriched organic formulations on yield attributes and yield of okra: **(A)** pods per plant, **(B)** pod length, **(C)** fruits per plant, **(D)** average fruit weight, **(E)** fruit yield, and **(F)** stover yield during 2021 and 2022 at harvest. The data represent the mean values across treatments (T_1_–T_7_) with error bars indicating standard deviation. Statistical significance was assessed using one-way ANOVA, with differences considered significant at *p* ≤ 0.05. Similar letter indicates the treatments are at par with each other at *p* ≤ 0.05.

In okra crop, yield attributes such as days to 50% flowering, days to first harvest, and average weight of fruit was found non-significantly different when applied with different enriched organic formulations. Yield attributing character, fruits per plant varied significantly (*p* ≤ 0.05) with the application of enriched organic formulations, demonstrated significantly higher values with T_4_ (11.5 and 12.0) in contrast to control treatment (T_1_) during 2021 and 2022. This treatment exhibited statistically parity with T_6_ and T_2_ during the years 2021 and 2022. The okra fruit (13.54 and 13.78 t ha^−1^) and stover yield (21.64 and 22.03 t ha^−1^) was discerned significantly highest when subjected to treatment T_4_ and this increase was found to be comparable to the yields achieved with T_6_ and T_2_ during both the years (2021 and 2022). Application of T_4_ caused a notable augmentation of fruit yield by 58.6% during 2021 and 59.2% during 2022 over control. In addition, T_5_ at parity with T_7_ also registers 28.2 and 29.5% higher fruit yield over control, respectively, during both the years (**Figures 6A–F**).

### Changes in soil physico-chemical and biological properties

No significant variation in the pH and BD was recorded when applied with different enriched organic formulations. However, lowest pH (7.73) and BD (1.48) were observed under the treatment T_4_ and statistically comparable with other treatments. Similarly, lowest EC (0.328 ds m^−1^) recorded under T_4_ and found significantly superior over T_5_, T_7,_ and T_1_. The treatment T_4_ (53.7%) closely followed by T_6_ (52.8%) and T_2_ (52.5%) showed significant (*p* ≤ 0.05) supremacy in rising the water-stable aggregates in soil by 15.7% over treatment T_1_ (control) as given in **Table 4**. The status of TOC at the end of two-year cropping cycle was remained similar under all the enriched organic formulations. However, highest organic carbon was obtained under T_4_ (0.41%) and lowest under control (0.37%).

**Table 4:** Effect of enriched organic formulations on soil physio-chemical properties, total organic carbon, MBC and microbial community composition in soil at the end of two-year cropping cycle.

Treatment	pH	EC	B.D. (Mg m^-3^)	WSA (%)	TOC (%)	MBC (mg kg^−1^)	Bacteria (×10^5^ cfu)	Fungi (×10^2^ cfu)	Actinomycetes (×10^2^ cfu)
T_1_: Control	7.98 ± 0.09^b^	0.368 ± 0.01^b^	1.54 ± 0.02^d^	46.4 ± 0.01^d^	0.37 ± 0.01^c^	177.2 ± 4.0 ^d^	29.9 ± 1.0^d^	14.8 ± 0.3^c^	12.1 ± 0.2^c^
T_2_: FYM	7.80 ± 0.16^ab^	0.346 ± 0.01^ab^	1.49 ± 0.02^ab^	52.5 ± 0.01^abc^	0.40 ± 0.01^ab^	217.3 ± 2.6^ab^	35.2 ± 1.2^ab^	19.5 ± 1.1^a^	16.6 ± 0.6^a^
T_3_: Improved RRC	7.88 ± 0.11^ab^	0.351 ± 0.02^ab^	1.50 ± 0.01^bc^	49.9 ± 0.02^abcd^	0.39 ± 0.02^abc^	208.0 ± 8.0^bc^	33.4 ± 1.3^bc^	17.4 ± 0.5^b^	14.7 ± 0.2^b^
T_4_: PHA-F (100% RDN)	7.73 ± 0.06^a^	0.328 ± 0.01^a^	1.48 ± 0.02^a^	53.7 ± 0.01^a^	0.41 ± 0.01^a^	227.7 ± 3.6^a^	36.2 ± 1.2^a^	20.3 ± 0.7^a^	17.5 ± 0.2^a^
T_5_: PHA-F (75% RDN)	7.92 ± 0.05^ab^	0.359 ± 0.01^b^	1.51 ± 0.01^c^	49.6 ± 0.02^bcd^	0.39 ± 0.02^abc^	199.6 ± 6.9^c^	33.2 ± 0.6^bc^	17.2 ± 0.4^b^	14.5 ± 0.4^b^
T_6_: PPC-F (100% RDN)	7.77 ± 0.07^ab^	0.341 ± 0.01^ab^	1.49 ± 0.02^ab^	52.8 ± 0.01^ab^	0.40 ± 0.01^ab^	220.2 ± 11.3^ab^	35.5 ± 0.6^ab^	19.7 ± 0.6^a^	16.8 ± 0.6^a^
T_7_: PPC-F (75% RDN)	7.93 ± 0.12^ab^	0.364 ± 0.01^b^	1.52 ± 0.02^cd^	48.8 ± 0.01^cd^	0.38 ± 0.01^bc^	193.5 ± 4.4^cd^	32.5 ± 1.2^cd^	16.4 ± 0.4^b^	13.7 ± 0.7^b^
LSD (*p* ≤ 0.05)	NS	0.024	NS	3.6	NS	15.0	2.4	1.4	1.1

NS indicate treatment is non-significant.

Significant variation in MBC, total organic carbon (TOC), and microbial population in soil after completion of two-years cropping cycle had been observed under different enriched organic formulations as depicted in **Table 4**. Application of treatment T_4_ registered significantly (*p* ≤ 0.05) highest value of MBC (227.7 µg C g^−1^ soil) to the extent of 28.5% over control followed by T_6_ (220.2 µg C g^−1^ soil) and T_2_ (217.3 µg C g^−1^ soil) at the end of two-year cropping cycle. Furthermore, treatment T_4_ demonstrated significantly (*p* ≤ 0.05) higher population counts of bacteria (36.2 ×10^5^ cfu), fungi (20.3 ×10^2^ cfu), and actinomycetes (17.5 × 10^2^ cfu) followed by T_6_ and T_2_. Treatment T_4_ recorded an increase of 21.1% population counts of bacteria, 37.2% counts of fungi, and 44.6% counts of actinomycetes over control at the end of two years cropping cycle. However, the lowest microbial population counts of bacteria (29.9 ×10^5^ cfu), fungi (14.8 ×10^2^ cfu), and actinomycetes (12.1 ×10^2^ cfu) was found under control treatment (T_1_).

Application of all enriched organic formulations improved the soil DHA and AP by 11.6%–43.8% and 9.9%–45.6% relative to the control at 50% flowering stage in all the crops. Treatment T_4_ performed equivalently to T_6_ and T_2_ exhibited significantly (*p* ≤ 0.05) heightened activity of dehydrogenase (210.1 and 207.8 μg TPF g^−1^ soil day^−1^) in pigeon pea, (206.4 and 208.4 μg TPF g^−1^ soil day^−1^) in vegetable mustard and (204.0 and 206.7 μg TPF g^−1^ soil day^−1^) in okra over control treatment (T_1_). The AP (80.2 and 82.4 μg PNP g^−1^ soil hr^−1^) in pigeon pea, (76.9 and 81.3 μg PNP g^−1^ soil hr^−1^) in veg mustard, and (84.1 and 89.2 μg PNP g^−1^ soil hr^−1^) in okra found significantly higher in treatment T_4_, which was comparable with Treatment T_6_ and T_2_ in soil at 50% flowering of all the crops in comparison to control at both the years (2020–2021 and 2022). The lowest values of DHA were recorded in pigeon pea (149.4 ± 4.8 and 149.6 ± 3.6 μg TPF g^−1^ soil day^−1^), vegetable mustard (147.4 ± 4.3 and 145.6 ± 4.1 μg TPF g^−1^ soil day^−1^) and okra (145.1 ± 6.0 and 143.7 ± 3.7 μg TPF g^−1^ soil day^−1^) under treatment T_1_. Similarly, the lowest values of AP were recorded in pigeon pea (58.4 ± 1.9 and 60.8 ± 1.8 μg TPF g^−1^ soil day^−1^), vegetable mustard (56.4 ± 2.0 and 57.3 ± 4.7 μg TPF g^−1^ soil day^−1^) and okra (54.7 ± 1.7 and 55.0 ± 4.3 μg TPF g^−1^ soil day^−1^) under treatment T_1_ (**Table 5**).

**Table 5:** Effect of enriched organic formulations on soil DHA and AP phosphatase activity at 50% flowering of pigeon pea–veg mustard–okra cropping system.

Treatment	DHA (μg TPF g^−1^ soil day^−1^)						AP (μg PNP g^−1^ soil hr^−1^)					
	Pigeon pea		Veg mustard		Okra		Pigeon pea		Veg mustard		Okra	
	2020	2021	2020–2021	2021–2022	2021	2022	2020	2021	2020–2021	2021–2022	2021	2022
T_1_: Control	149.4 ± 4.8^d^	149.6 ± 3.6^d^	147.0 ± 4.3^c^	145.8 ± 4.1^e^	145.1 ± 6.0^c^	143.7 ± 3.7^e^	58.4 ± 1.9^e^	60.8 ± 1.8^d^	56.4 ± 2.0^e^	57.3 ± 4.7^d^	54.7 ± 1.7^d^	55.0 ± 4.3^d^
T_2_: FYM	196.6 ± 9.8^ab^	198.6 ± 6.1^a^	194.6 ± 7.7^a^	197.7 ± 4.8^b^	191.5 ± 5.8^a^	194.3 ± 3.4^b^	75.1 ± 3.0^ab^	78.0 ± 2.0^a^	72.8 ± 2.5^ab^	76.8 ± 2.1^a^	70.8 ± 2.0^a^	75.5 ± 1.0^a^
T_3_: Improved RRC	182.6 ± 5.8^bc^	182.3 ± 4.4^b^	180.2 ± 5.5^b^	179.1 ± 4.9^c^	177.4 ± 6.4^b^	177.1 ± 6.5^c^	70.6 ± 0.5^bc^	72.9 ± 1.5^b^	68.6 ± 0.4^bc^	70.3 ± 1.9^b^	66.5 ± 1.1^b^	68.6 ± 2.2^b^
T_4_: PHA-F (100% RDN)	210.1 ± 4.9^a^	207.8 ± 2.0^a^	206.4 ± 3.9^a^	208.4 ± 4.0^a^	204.0 ± 4.5^a^	206.7 ± 3.5^a^	80.2 ± 2.4^a^	82.4 ± 1.7^a^	76.9 ± 0.7^a^	81.3 ± 1.3^a^	74.9 ± 1.8^a^	80.1 ± 0.6^a^
T_5_: PHA-F (75% RDN)	177.9 ± 4.6^c^	181.6 ± 4.5^b^	176.3 ± 3.3^b^	178.6 ± 4.7^c^	173.8 ± 4.4^b^	176.7 ± 4.6^c^	69.0 ± 1.9^cd^	72.1 ± 1.8^b^	67.0 ± 2.5^c^	69.9 ± 1.6^b^	65.0 ± 2.0^b^	68.3 ± 2.1^b^
T_6_: PPC-F (100% RDN)	199.8 ± 11.6^a^	199.3 ± 7.8^a^	196.5 ± 9.8^a^	198.0 ± 6.6^b^	193.4 ± 9.5^a^	193.2 ± 10.5^b^	76.4 ± 2.4^a^	78.8 ± 2.7^a^	74.0 ± 2.6^a^	77.4 ± 2.7^a^	71.9 ± 3.0^a^	75.9 ± 2.1^a^
T_7_: PPC-F (75% RDN)	168.8 ± 2.7^c^	166.9 ± 1.3^c^	166.5 ± 2.3^b^	162.9 ± 1.2^d^	163.7 ± 2.7^b^	160.5 ± 1.7^d^	64.5 ± 3.1^d^	65.9 ± 1.7^c^	62.0 ± 0.9^d^	62.3 ± 1.6^c^	60.5 ± 0.6^c^	60.8 ± 1.8^c^
LSD (*p* ≤ 0.05)	16.0	10.5	13.3	9.9	13.7	12.0	5.3	4.5	4.3	4.8	4.1	5.2

Similar letter indicates the treatments are at par with each other at *p* ≤ 0.05.

The mineral nitrogen (NH_4_-N and NO_3_-N) content in soil at harvest of crops under various enriched organic formulations are depicted in **Table 6**. Among these formulations, treatment T_4_ demonstrated significantly (*p* ≤ 0.05) higher values of NH_4_-N (10.2 ± 0.3 and 10.4 ± 0.2 mg kg^−1^) and NO_3_-N (18.6 ± 0.8 and 20.4 ± 0.7 mg kg^−1^) content in soil after harvest of okra followed by application of T_6_ (9.9 ± 0.3 and 9.9 ± 0.3 mg kg^−1^ NH_4_-N and 18.1 ± 0.1 and 19.8 ± 0.6 mg kg^−1^ NO_3_-N) and T_2_ (9.7 ± 0.2 and 9.8 ± 0.1 mg kg^−1^ NH_4_-N and 17.9 ± 0.3 and 19.7 ± 0.5 mg kg^−1^ NO_3_-N) and consequently this treatment was superior over rest of the treatments with respective increase of NH_4_-N by 72.9% and 112.2%, NO_3_-N by 124.1% and 158.2% over control treatment (T_1_).

**Table 6:** Effect of enriched organic formulations on soil NH_4_-N and NO_3_-N, available N, P, and K status of soil at harvest of pigeon pea–veg mustard–okra cropping system.

Treatment	Soil NH_4_-N (mg kg^−1^)		Soil NO_3_-N (mg kg^−1^)		Available N (kg ha^−1^)		Available P (kg ha^−1^)		Available K (kg ha^−1^)	
	2020–21	2021–22	2020–21	2021–22	2020–21	2021–22	2020–21	2021–22	2020–21	2021–22
T_1_: Control	5.9 ± 0.2^d^	4.9 ± 0.2^d^	8.3 ± 0.3^d^	7.9 ± 0.3^d^	199.9 ± 3.1^d^	192.9 ± 2.3^d^	10.6 ± 0.4 ^ac^	9.6 ± 0.5^e^	230.3 ± 5.4^c^	224.1 ± 4.7^d^
T_2_: FYM	9.7 ± 0.2^a^	9.8 ± 0.1^a^	17.9 ± 0.3^a^	19.7 ± 0.5^a^	222.4 ± 2.8^abc^	222.4 ± 3.4^ab^	15.0 ± 0.3^b^	14.6 ± 0.2^bc^	243.3 ± 4.9^bc^	244.9 ± 4.4^bc^
T_3_: Improved RRC	8.8 ± 0.2^b^	8.9 ± 0.1^b^	15.6 ± 0.5^b^	17.1 ± 0.1^b^	216.3 ± 4.1^abc^	216.4 ± 2.6^abc^	14.0 ± 0.8^b^	13.7 ± 0.6^cd^	262.2 ± 8.0^a^	263.7 ± 8.6^a^
T_4_: PHA-F (100% RDN)	10.2 ± 0.3^a^	10.4 ± 0.2^a^	18.6 ± 0.8^a^	20.4 ± 0.7^a^	224.9 ± 7.8^ab^	225.8 ± 7.2^ab^	15.6 ± 0.2^a^	15.6 ± 0.5^a^	255.2 ± 12.9^ab^	256.6 ± 12.1^ab^
T_5_: PHA-F (75% RDN)	8.7 ± 0.5^b^	8.8 ± 0.5^b^	15.3 ± 0.3^b^	16.9 ± 0.4^b^	213.0 ± 5.9^c^	210.3 ± 6.2^c^	13.9 ± 0.1^b^	13.5 ± 0.1^d^	243.0 ± 6.2^bc^	244.7 ± 4.5^bc^
T_6_: PPC-F (100% RDN)	9.9 ± 0.3^a^	9.9 ± 0.3^a^	18.1 ± 0.1^a^	19.8 ± 0.6^a^	227.2 ± 2.3^a^	227.3 ± 4.1^a^	15.1 ± 0.4^a^	14.8 ± 0.4^ab^	247.3 ± 6.1^abc^	251.1 ± 5.1^abc^
T_7_: PPC-F (75% RDN)	7.4 ± 0.2^c^	7.4 ± 0.1^c^	14.0 ± 0.2^c^	15.8 ± 0.3^c^	215.1 ± 4.2^ab^	215.1 ± 4.9^bc^	13.4 ± 0.4^b^	13.6 ± 0.5^d^	237.4 ± 5.3^bc^	238.1 ± 4.2^cd^
LSD (*p* ≤ 0.05)	0.7	0.6	1.0	1.1	10.9	10.7	0.8	0.9	17.2	15.5

Similar letter indicates the treatments are at par with each other at *p* ≤ 0.05.

The availability of soil N, P, and K varied substantially under different enriched organic formulations compared to control treatment (T_1_) after harvest of pigeon pea-vegetable mustard-okra cropping system as depicted in **Table 6**. Application of T_6_ demonstrated substantially (*p* ≤ 0.05) enhanced amount of available N in soil after harvest of okra (227.2 ± 2.3 and 227.3 ± 4.1 kg ha^−1^) crop over T_1_ treatment (199.9 ± 3.1 and 192.9 ± 2.3 kg ha^−1^) and the extent of increase was 13.7% during first year and 17.8% during second year and exhibited statistically parity with other organic formulations. Application of T_4_ being comparable to T_6_ and T_2_ observed greater amount of available P (15.6 ± 0.2 and 15.6 ± 0.5 kg ha^−1^) in soil after harvest of okra crop and found significantly (*p* ≤ 0.05) superior over T_3_ (14.0 ± 0.8 and 13.7 ± 0.6 kg ha^−1^), T_5_ (13.9 ± 0.1 and 13.5 ± 0.1 kg ha^−1^), T_7_ (13.4 ± 0.4 and 13.6 ± 0.5 kg ha^−1^), and control (10.6 ± 0.4 and 9.6 ± 0.5 kg ha^−1^) by 11.4%, 12.2%, 16.4%, and 47.2% during first year and 13.9%, 15.6%, 14.7%, and 62.5% during second year respectively. Furthermore, treatment T_3_ (262.2 ± 8.0 and 263.7 ± 8.6 kg ha^−1^) remaining on par with T_4_ (255.2 ± 12.9 and 256.6 ± 12.1 kg ha^−1^) and T_6_ (247.3 ± 6.1 and 251.1 ± 5.1 kg ha^−1^) exhibited a noteworthy increment in available K content in soil over control by 13.9% during 2020–21 and 17.7% during 2021–22 after harvest of okra crop. Further, the apparent N budget showed that the highest negative N balance under control treatment (T_1_) after completion of two cropping cycles. Among the treatments applied with enriched organic formulations, T_4_ and T_6_ recorded a negative balance of 46.4 and 31.2 kg N ha^−1^, respectively, after completion of experiment. The treatment T_3_ showed maximum positive balance of 98.9 kg N ha^−1^ followed by treatment T_5_ and T_7_ (**Supplementary Table 1**).

#### Correlation between organic carbon added and soil parameters at the end of two cropping cycles

**Figure 7:**
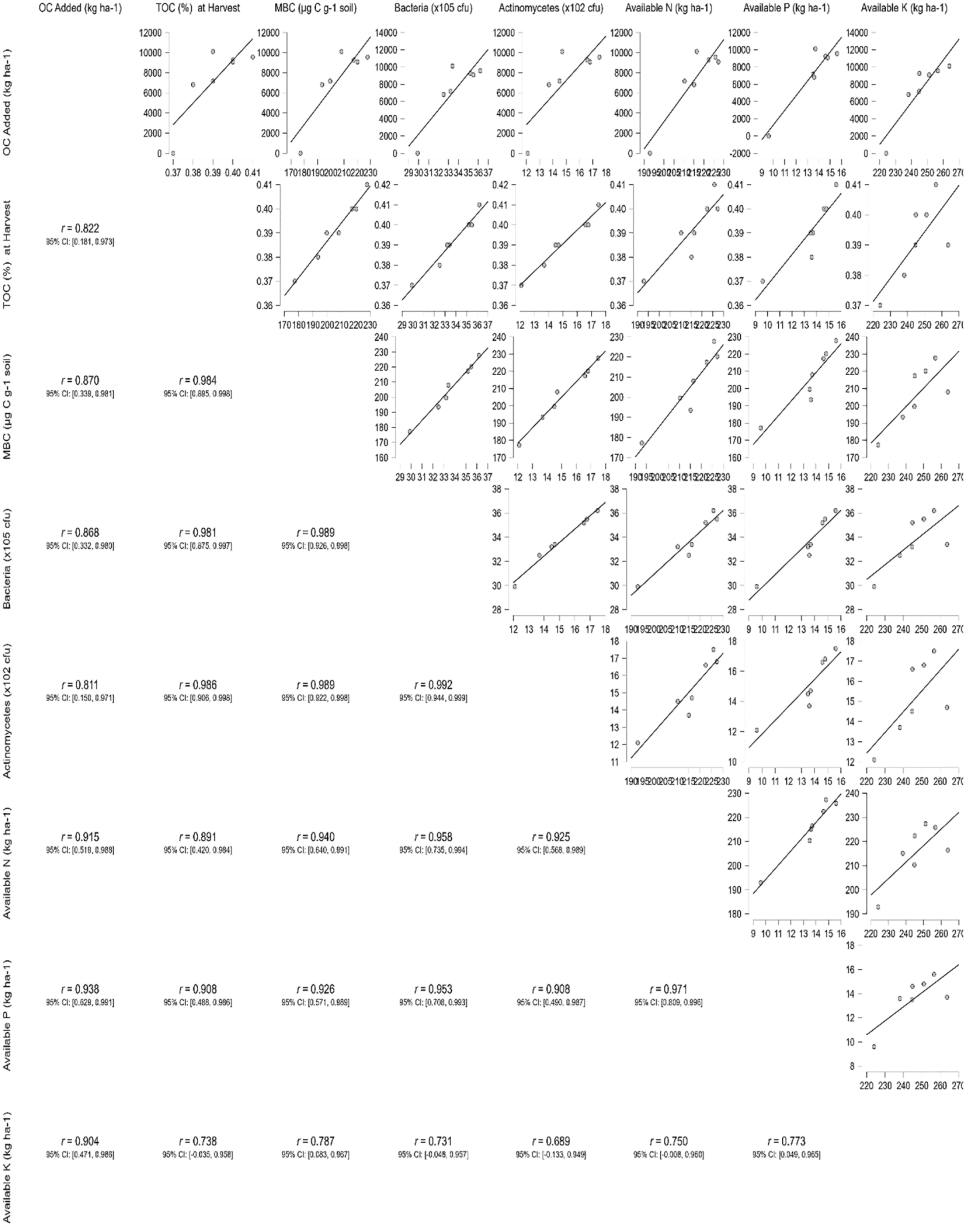
Correlation matrix of organic carbon added via enriched formulations with total organic carbon, microbial population, and nutrient availability in soil at the end of two cropping cycles.

The correlation analysis depicted in the matrix plot (**Figure 7**; **Supplementary Table 1**) revealed notable positive associations (> 0.822) between the amount of organic carbon added through enriched formulations and the TOC content measured at harvest. Similarly, strong positive correlations (*r* > 0.811) were observed between the added organic carbon and MBC and microbial counts. Furthermore, substantial positive relationships (*r* > 0.731) were identified between the added organic carbon, TOC, MBC, microbial counts, and nutrient availability measured at the conclusion of two cropping cycles. These findings highlight the interconnected nature of organic carbon inputs and various soil parameters, emphasizing their influence on soil carbon content, microbial activity, and nutrient availability over the experimental period.

## Discussion

In present study, the impact of enriched organic formulations on growth parameters was discernible at later stages of crop growth contrast to the treatment T_1_ in both 2020 and 2021. The heightened growth observed in the treatment T_4_ can be attributed to escalated activities of beneficial microorganisms, facilitated by the augmented organic pool in the soil resulting from the application of nutrient sources in pigeon pea–vegetable mustard–okra cropping system. Moreover, the improved growth observed in treatments T_2_ and T_6_ can be attributed to the beneficial effects of organic sources. These effects are likely due to the impact of soil organic matter (SOM), which enhances the physical, chemical, and biological properties of the soil, creating a favorable environment for better plant growth in pigeon pea–vegetable mustard-okra cropping system. Furthermore, the presence of humic acids released during the decomposition of organic materials enhances the availability of soil nutrients, leading to improved growth parameters (Tiwari et al., 2023).

Yield attributes are mainly influenced by genetic characteristics of varieties and environmental factors. Treatment T_4_ recorded highest increase in yield attributes and yield (seed and stover) comparable to T_6_ and T_2_ over treatment T_1_ during 2020–2021 and 2021–2022, respectively. Organic manures serve as a reservoir of nutrients, producing organic acids during decomposition. These acids gradually release absorbed ions throughout the crop’s entire growth cycle, leading to improved yield attributes and yield under pigeon pea–vegetable mustard–okra cropping system. Additionally, the increase in yield attributes observed in treatment, T_2_, T_4_, and T_6_ can be attributed to an increase in photosynthetic area, DMA and translocation of photosynthates toward sink tissues. The significant improvement in seed and stover yield resulting from the use of organic nutrient sources could be attributed their beneficial impact on yield attributes and their combined influence, which primarily contribute to enhanced productivity. Pradeep et al. (2018) reported increased yield attributing characters in pigeon pea when supplemented with 100% RDN through vermi-compost (2 t ha^−1^) along with FYM @7.5 t ha^−1^ as compared to other treatments. Findings of study done by Jakhro et al. (2017) reported that 75 kg N + 6 tons FYM ha^−1^ resulted in higher fresh weight per plant, leaf length, and leaf yield of spinach over control. In current study, no significant difference was observed in pH and BD, but the lowest pH and BD were observed under T_4_ treatment and noticeably comparable to other treatments applied with difference enriched organic formulations. The reduction in pH caused by addition of organic manure might be due to production of organic acid during breakdown of SOM, which dissolves native salts. In treatments applied with enriched organic formulations recorded increase in BD and water-stable aggregates. The use of organic nutrient sources resulted in a decrease in BD due to increased soil organic carbon (SOC) content and enhanced root biomass. The positive effects of certain polysaccharides formed during the breakdown of organic waste by microbial activity as well as the cementing action of bacteria and fungi may be the cause of rise in water-stable aggregates in the FYM treated plot. This, in turn, facilitated improved soil aeration and better soil aggregation. Nandapure et al. (2011) reported that FYM application @5t ha^−1^ in sorghum-wheat cropping system recorded enhanced percentage of WSAs by 11.1% than unfertilized control plot. Gupta et al. (2012) also observed that FYM use @5t ha^−1^ did not bring substantial difference in pH and EC of soil and remain at par with remaining treatments after complete rotation of maize-wheat cropping system.

Treatment T_4_ registered significantly (*p* ≤ 0.05) highest value of MBC to the extent of 28.5% over control (T_1_) and found at par with T_6_ and T_2_ at the end of two-year cropping cycle. The addition of readily decomposable carbon sources act as important sources of energy to carry out the activities of microorganisms, soil enzymes, and MBC. The increase in MBC in treatment T_4_, T_6,_ and T_2_ may be attributed to application of organic carbon through enriched organic formulations. Almeida et al. (2011) found that adding organic carbon to the soil from nutrient sources was closely linked to increased microbial and enzymatic activity, showing a positive relationship with MBC. Kaur et al. (2005) reported that organic manures promotes the MBC in soil whereas, Gong et al. (2009) found an increase in enzymatic activities due to addition of organic manures as compared to mineral fertilizers. The status of SOC at the end of two cropping cycle was remained non-significant under all the treatments. However, highest organic carbon was obtained under T_4_ (0.41%) due to increase application of organic carbon through application of enriched organic formulations. Gupta et al. (2012) reported that FYM application @ 5 t ha^−1^ did not bring substantial variation in SOC and exhibiting statistical parity with remaining treatments at the end of maize–wheat cropping system. The DHA and AP activity was recorded significantly superior in all the treatment applied with enriched organic formulations over control (T_1_) in all the crops. The highest DHA and AP values were recorded in T_4_, which was comparable with T_2_ and T_6_. The activities of DHA and AP were significantly affected by the amount of organic matter added to soils through various applications: 100% RDN via PHA/PPC-based formulation, 100% RDN via FYM, 100% RRC, and 75% RDN via PHA/PPC-based formulation. These formulations contain easily decomposable organic carbon components that exert a significant impact on the metabolic activity of soil microorganisms, resulting in enhanced DHA and AP activity in the soil. Applying organic source of nutrients improved the organic carbon which was correlated with higher enzymatic activity (Mandal et al., 2007).

Among different formulations, treatment T_4_ demonstrated significant (*p* ≤ 0.05) increased values of NH_4_-N and NO_3_-N content in soil over control (T_1_) after harvest of okra during 2020–21 and 2021–22 and also found on par with treatment T_6_ and T_2_. The higher mineral nitrogen content in soil under different treatment compared to control (T_1_) may be due to enhanced rate of SOM mineralization in the soil which was further enhanced by the addition of enriched organic formulation, resulted in build-up of NH_4_-N and NO_3_-N in soil. Bhardwaj et al. (2023) reported that addition of organic manure along with fertilizers enhance the mineralization thereby increase the NH_4_-N and NO_3_-N in soil.

All the treatment with enriched organic formulations showed parity among each for available soil nutrient content and found significantly superior over control (T_1_). Treatment T_6_ demonstrated significantly (*p* ≤ 0.05) highest amount of available N in soil after harvest of okra crop over control (T_1_). The increased availability of nitrogen in the soil could be attributed to the favorable soil conditions fostered by the use of enriched organic formulations. These conditions likely facilitated nitrogen mineralization, resulting in higher accumulation of available nitrogen. The addition of organic matter through enriched organic formulations increased Olsen-P due to its P content and perhaps by enhancing P retention in soil through release of different organic acids and CO_2_ during the decomposition of SOM. Further, enriched organic formulations increased soil cation exchange capacity and decreased K fixation in soil, the increase in available K under these treatments was attributed to higher release of non-exchangeable K from the soils. The direct introduction of N, P, and K either independently or through the simultaneous application of organic nutrient sources contributes to an active soil pool, resulting in increased overall N availability as well as enhanced availability of N, phosphorus P, and K in the soil (Babu et al., 2020; Nima et al., 2020). Further, the apparent N budget showed that the highest negative N balance under control treatment (T_1_) after completion of two cropping cycles due to no application of any fertilizer or organic source of nutrient. Among the treatments applied with enriched organic formulations, T_4_ and T_6_ recorded an apparent negative balance of 46.4 and 31.2 kg N ha^−1^ respectively which might be attributed to increased nutrient uptake and enhanced yield. The treatment T_3_ showed maximum positive balance of 98.9 kg N ha^−1^ followed by treatment T_5_ and T_7_ which could be attributed to lower yield and nutrient uptake comparison to treatment T_4_ and T_6_.

In our study, correlation analysis showed that the relationships between organic carbon additions, soil properties, microbial biomass, and nutrient availability. The findings from this analysis provide valuable insights into the impact of organic inputs on soil health and nutrient dynamics. Firstly, we observed significant positive correlations (> 0.822) between the amount of organic carbon added through enriched formulations and the TOC content at harvest. This suggests that the application of enriched organic materials contributes to an increase in SOC levels over the course of the cropping cycles. Secondly, strong positive associations (*r* > 0.811) were identified between the added organic carbon and MBC and microbial counts. This indicates that the introduction of organic amendments promotes microbial activity in the soil. Furthermore, our analysis revealed substantial positive relationships (*r* > 0.731) among organic carbon additions, TOC, MBC, microbial counts, and nutrient availability at the end of the two cropping cycles. These findings highlight the interconnected nature of soil parameters influenced by organic inputs, suggesting that enriched organic formulations can positively impact soil health and nutrient dynamics. Overall, these correlations underscore the importance of incorporating enriched organic materials in agricultural practices to promote soil carbon sequestration, microbial activity, and nutrient availability. The observed relationships provide scientific support for the use of sustainable organic amendments to optimize agricultural productivity and environmental stewardship.

## Conclusion

The application of 100% RDN through PHA and PPC based formulations proved to be optimal for achieving enhanced growth and yield in the pigeon pea–vegetable mustard–okra cropping system. These formulations exhibited comparable results to the traditional method of applying 100% RDN through FYM. Notably, the application of 100% RDN through PHA-based formulation emerged as particularly effective in enhancing soil quality. This effectiveness is evident in the significantly elevated activities of dehydrogenase and AP enzymes, increased nutrient availability in the soil, elevated SOC levels, improved soil aggregation, and enhanced MBC and microbial populations at the harvest of the pigeon pea-based cropping system. Consequently, these formulations are recommended for adoption in regions facing a shortage of FYM but having access to rice husk ash and potato peels, providing a sustainable solution for utilizing agricultural wastes and advancing agricultural sustainability. The findings underscore the potential of these formulations to contribute significantly to improved agricultural practices, offering a harmonious blend of environmental sustainability and enhanced food security in areas experiencing FYM scarcity.

## Data availability statement

The original contributions presented in the study are included in the article/**Supplementary Material**. Further inquiries can be directed to the corresponding authors.

## Author contributions

KG: Investigation, Writing – original draft, Formal analysis. SD: Conceptualization, Formal analysis, Supervision, Writing – original draft, Writing – review & editing, Project administration, Validation. VS: Formal analysis, Visualization, Writing – original draft. EA: Visualization, Writing – original draft. RM: Data curation, Writing – original draft. MH: Formal analysis, Visualization, Writing – original draft. DK: Formal analysis, Methodology, Writing – original draft. GA: Data curation, Formal analysis, Writing – original draft. VK: Formal analysis, Visualization, Writing – review & editing. YK: Data curation, Writing – review & editing. SA: Formal analysis, Writing – original draft. SoK: Formal analysis, Writing – original draft. HO: Formal analysis, Writing – original draft. MT: Formal analysis, Writing – review & editing. BM: Visualization, Writing – review & editing, Formal analysis. BK: Data curation, Visualization, Writing – original draft. VM: Formal analysis, Writing – original draft. SaK: Data curation, Formal analysis, Methodology, Visualization, Writing – original draft, Writing – review & editing.
