# Supplementary material for: Optimizing agricultural sustainability: enriched organic formulations for growth, yield, and soil quality in a multi-crop system
Source: Front Plant Sci. 2024 Jun 19;15:1398083. doi: 10.3389/fpls.2024.1398083 (PMC11219940; doi:10.3389/fpls.2024.1398083)
Supplement: Supplementary file 1 [file DataSheet_1.pdf]

Supplementary Table 1. Effect of enriched organic formulations on Apparent N budgeting under Pigeon pea-Vegetable mustard-Okra cropping system.

Treatment	Available soil N (initial)	Input			Total N uptake	Available soil N (final)	Apparent N balance
		RDN	BNF	Total N			
	1	2	3	2+3=4	5	6	4-5=7
T_1: Control	206.2	0	340	340	441.6	192.9	-88.3
T_2: FYM	206.2	420	340	760	730.7	222.4	13.1
T_3: RRC	206.2	420	340	760	650.9	216.4	98.9
T_4: PHA-F (100% RDN)	206.2	420	340	760	786.8	225.8	-46.4
T_5: PHA-F (75% RDN)	206.2	315	340	655	622.3	210.3	28.6
T_6: PPC-F (100% RDN)	206.2	420	340	760	770.1	227.3	-31.2
T_7: PPC-F (75% RDN)	206.2	315	340	655	616.1	215.1	30

Supplementary Table 2: Correlation matrix of organic carbon added via enriched formulations with total organic carbon, microbial population, and nutrient availability in soil at the end of two cropping cycles.

		Pearson's Correlations							
**Variable**		**OC Added (kg ha^{-1})**	**TOC (%) at Harvest**	**MBC (µg C g^{-1} soil)**	**Bacteria (x10^{5} cfu)**	**Actinomycetes (x10^{2} cfu)**	**Available N (kg ha^{-1})**	**Available P (kg ha^{-1})**	**Available K (kg ha^{-1})**
1. OC Added (kg ha-1)	Pearson's r	—							
	p-value	—							
	Upper 95% CI	—							
	Lower 95% CI	—							
2. TOC (%) at Harvest	Pearson's r	0.822 *	—						
	p-value	0.023	—						
	Upper 95% CI	0.973	—						
	Lower 95% CI	0.181	—						
3. MBC (µg C g^{-1} soil)	Pearson's r	0.870 *	0.984 ***	—					
	p-value	0.011	< .001	—					
	Upper 95% CI	0.981	0.998	—					
	Lower 95% CI	0.339	0.895	—					
4. Bacteria (x10^{5} cfu)	Pearson's r	0.868 *	0.981 ***	0.989 ***	—				
	p-value	0.011	< .001	< .001	—				
	Upper 95% CI	0.980	0.997	0.998	—				
	Lower 95% CI	0.332	0.875	0.926	—				
5. Actinomycetes (x10^{2} cfu)	Pearson's r	0.811 *	0.986 ***	0.989 ***	0.992 ***	—			
	p-value	0.027	< .001	< .001	< .001	—			
	Upper 95% CI	0.971	0.998	0.998	0.999	—			
	Lower 95% CI	0.150	0.906	0.922	0.944	—			
6. Available N (kg ha^{-1})	Pearson's r	0.915 **	0.891 **	0.940 **	0.958 ***	0.925 **	—		
	p-value	0.004	0.007	0.002	< .001	0.003	—		
	Upper 95% CI	0.988	0.984	0.991	0.994	0.989	—		
	Lower 95% CI	0.519	0.420	0.640	0.735	0.568	—		
7. Available P (kg ha^{-1})	Pearson's r	0.938 **	0.908 **	0.926 **	0.953 ***	0.908 **	0.971 ***	—	
	p-value	0.002	0.005	0.003	< .001	0.005	< .001	—	
	Upper 95% CI	0.991	0.986	0.989	0.993	0.987	0.996	—	
	Lower 95% CI	0.629	0.488	0.571	0.708	0.490	0.809	—	
8. Available K (kg ha^{-1})	Pearson's r	0.904 **	0.738	0.787 *	0.731	0.689	0.750	0.773 *	—
	p-value	0.005	0.058	0.036	0.062	0.087	0.052	0.041	—
	Upper 95% CI	0.986	0.958	0.967	0.957	0.949	0.960	0.965	—
	Lower 95% CI	0.471	-0.035	0.083	-0.048	-0.133	-0.008	0.049	—

* p < .05, ** p < .01, *** p < .001
